# Supplementary material for: Risk factors for sacrococcygeal pilonidal sinus: a systematic review and meta-analysis supplemented by genetic causal assessment
Source: Front Surg. 2026 Jan 7;12:1718589. doi: 10.3389/fsurg.2025.1718589 (PMC12819706; doi:10.3389/fsurg.2025.1718589)
Supplement: Supplementary file 2 [file Datasheet2.zip › Supplementary Data 2/MR_pipeline_p5e-8/leaveone_plots/ukb-d-1747_3.finngen_R12_L12_PILONIDALCYST.pdf]

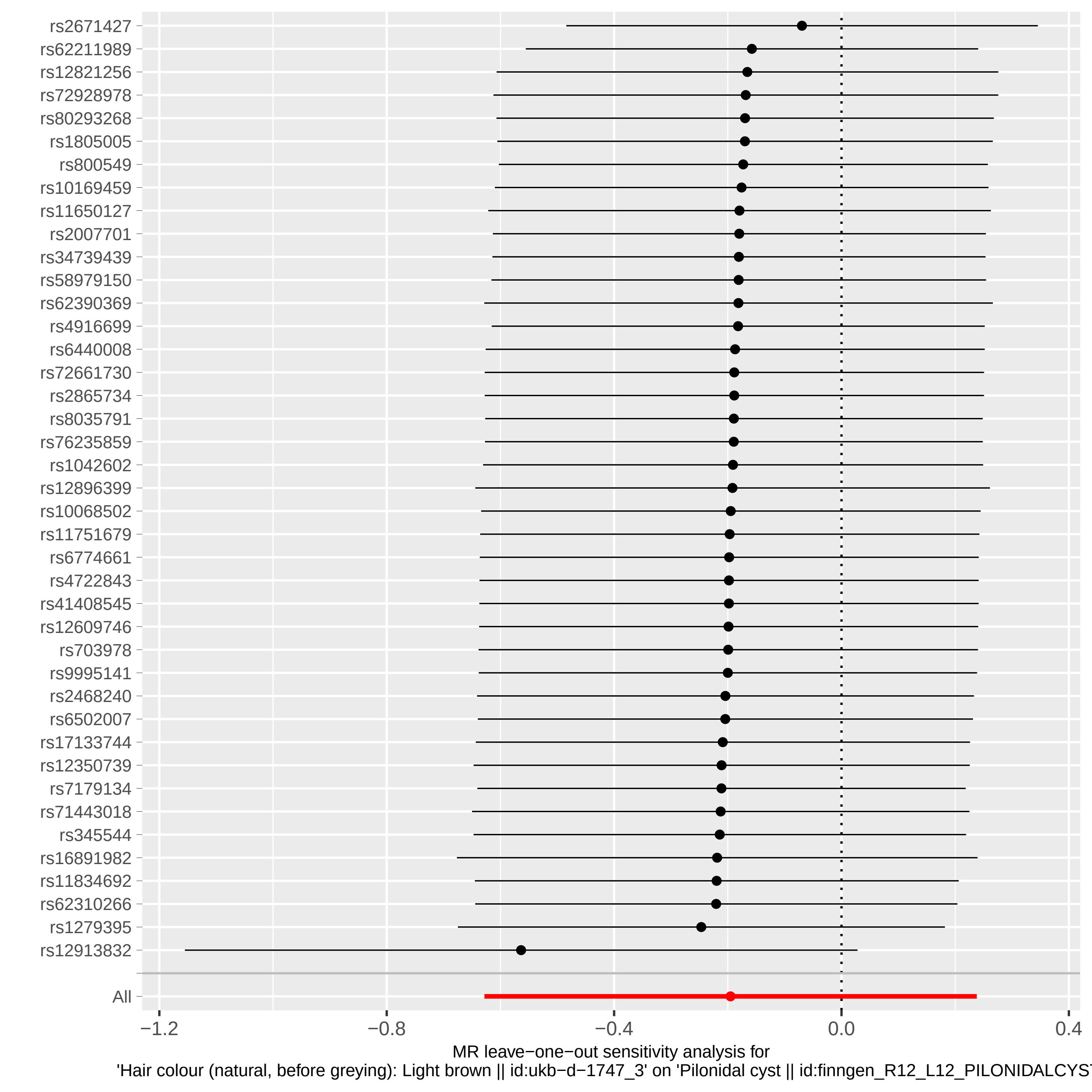
rs2671427
rs62211989
rs12821256
rs72928978
rs80293268
rs1805005
rs800549
rs10169459
rs11650127
rs2007701
rs34739439
rs58979150
rs62390369
rs4916699
rs6440008
rs72661730
rs2865734
rs8035791
rs76235859
rs1042602
rs12896399
rs10068502
rs11751679
rs6774661
rs4722843
rs41408545
rs12609746
rs703978
rs9995141
rs2468240
rs6502007
rs17133744
rs12350739
rs7179134
rs71443018
rs345544
rs16891982
rs11834692
rs62310266
rs1279395
rs12913832
All
−1.2
−0.8
−0.4
0.0
0.4
MR leave−one−out sensitivity analysis for
'Hair colour (natural, before greying): Light brown || id:ukb−d−1747_3' on 'Pilonidal cyst || id:finngen_R12_L12_PILONIDALCYS
